# Influence of Anodizing Parameters on Surface Morphology and Surface-Free Energy of Al_2_O_3_ Layers Produced on EN AW-5251 Alloy

**DOI:** 10.3390/ma12050695

**Published:** 2019-02-27

**Authors:** Marek Bara, Mateusz Niedźwiedź, Władysław Skoneczny

**Affiliations:** Department of Surface Layers Technology, Faculty of Computer Science and Materials Science, University of Silesia in Katowice, 40-007 Katowice, Poland; marek.bara@us.edu.pl (M.B.); mateusz.niedzwiedz@us.edu.pl (M.N.)

**Keywords:** aluminum oxide layers, nanomorphology, surface-free energy

## Abstract

The paper presents the influence of the surface anodizing parameters of the aluminum alloy EN AW-5251 on the physicochemical properties of the oxide layers produced on it. Micrographs of the surface of the oxide layers were taken using a scanning electron microscope (SEM). The chemical composition of cross-sections from the oxide layers was studied using energy dispersive spectroscopy (EDS). The phase structure of the Al_2_O_3_ layers was determined by the pattern method using X-ray diffractometry (XRD). The nanomorphology of the oxide layers were analyzed based on microscopic photographs using the ImageJ 1.50i program. The energetic state of the layers was based on the surface-free energy (SFE), calculated from measurements of contact angles using the Owens-Wendt method. The highest surface-free energy value (49.12 mJ/m^2^) was recorded for the sample produced at 293 K, 3 A/dm^2^, in 60 min. The lowest surface-free energy value (31.36 mJ/m^2^) was recorded for the sample produced at 283 K, 1 A/dm^2^, in 20 min (the only hydrophobic layer). The highest average value nanopore area (2358.7 nm^2^) was recorded for the sample produced at 303 K, 4 A/dm^2^, in 45 min. The lowest average value nanopore area (183 nm^2^) was recorded for the sample produced at 313 K, 1 A/dm^2^, in 20 min.

## 1. Introduction

Aluminum alloys are used increasingly in manufacturing engineering because of their favorable design parameters, good thermal conductivity and low price [1,2]. Aluminum alloys are used in the automotive, aerospace, and machine industries. The mechanical strength and corrosion protection of aluminum alloys are increased when oxide layers are formed on their surface. Usually, oxide layers are formed using the electrochemical anodic oxidizing process [3,4,5,6,7]. The essence of the electrochemical anodic oxidizing process is that the Al_2_O_3_ layers are formed at the expense of a loss of the aluminum alloy substrate. The result is an effective adhesion of the layers to the substrate. The most common method of oxidizing the surface of aluminum alloys is the DC anodizing process. At the beginning of the DC anodizing process, observations of the voltage show that initially the voltage increases rapidly to a maximum value, then decreases to a minimum value, and subsequently increases slowly. The anodizing voltage characteristics result from the process of destroying the natural barrier layer with high electrical resistance, and then constructing the porous layer [3,5,8,9]. The oxide layers exhibit a fibrous structure, formed in the direction of layer growth [3,5,10]. The fibers in the oxide layers are arranged parallel to each other, creating spaces between them. The porosity of the oxide layers determine their purpose. The type, size and shape of the pores depend on: the structure of the substrate, the conditions of etching the aluminum alloy surface, the type of electrolyte, and the conditions under which the anodizing process is carried out.

The surface of the Al_2_O_3_ layers contains: micropores, nanopores, as well as defects and distortions of the oxide structure. The micropores are formed as a result of disturbances in the structure caused by the transfer of substrate defects to the surface of the oxide layer and energy disturbances of the anodizing process [11]. The nanopores are formed as a result of the contact of the oxide fibers and are present in all the fiber cross-sections over the entire thickness of the oxide layers. A single nanopore cell contacts linearly with the six surrounding fibers, creating hexagonal channels [12]. The structure of the Al_2_O_3_ layers produced in electrolytes with a low-secondary dissolution capacity is very similar to the barrier layer construction. Oxide layers produced in electrolytes with high acid concentrations may take on a cross-section of a circle, oval, triangle or other polygon shape [13,14]. Distortions of the oxide structure result from the use of aluminum alloys with a large amount of alloy additives such as the substrate of the Al_2_O_3_ layers [11]. Distortions of the oxide substrate display fibers which are arranged in an irregular manner and at different angles. The alloy components occurring at the substrate-layer boundary cause local variations in the thickness of the oxide layer, induced by different anodizing speeds. When larger clusters of precipitates occur in the substrate at the layer formation border, layer growth is delayed, causing local reduction in the layer thickness and its defects [10]. The relatively regular distribution of high-density nanopores produce Al_2_O_3_ layers with good absorption properties. As a result, they are used as the matrix of composite layers [15]. The porosity of oxide layers is closely related to their energy state (surface-free energy), and the tendency of the material to repel and attract water molecules (hydrophobicity). Materials with hydrophobic features are highly sought after due to their extensive applications ranging from self-cleaning, through anti-icing to waterproofing [16,17]. Creating Al_2_O_3_ layers with hydrophobic properties would enable even more extensive applications [18]. Materials with hydrophilic properties are characterized by a low contact angle with the surface, and hence high wettability. The effects of process parameters on the properties of the oxide layers were investigated. The effects of varying the process parameters which have the greatest impact on changes in the surface morphology of the oxide layers were investigated. The process parameters which were varied included: Current density, process time, and electrolyte temperature. The range of process parameters was selected in such a way as to complete the previous tests [19].

## 2. Materials and Methods

### 2.1. Research Material

Al_2_O_3_ layers were produced on samples of the aluminum alloy EN AW-5251 with an area of 5 × 10^−4^ m^2^. The aluminum alloy EN AW-5251 is characterized by good strength properties with a small amount of alloy components and good susceptibility to anodizing. All the sample surfaces before the anodizing process were etched in a 5% KOH solution and then neutralized in a 10% HNO_3_ solution. The treatments ended with rinsing the sample surfaces in distilled water. The anodizing process was carried out in a ternary electrolyte solution consisting of 18% acid sulfuric (33 mL/L), oxalic (30 g/L), and phthalic acids (76 g/L) During the anodizing process the solution was stirred vigorously. The addition of C_8_H_6_O_4_ acid ensured a hard oxide layer and allowed the anodizing process to be carried out at room temperature. This reduced the cost of intensive electrolyte cooling during the anodizing process. Table 1 shows the conditions for anodizing the oxide layers.

The production of Al_2_O_3_ layers on the aluminum alloys was carried out in the electrolytic anodizing process using the direct current method by means of a stabilized GPR-25H30D power supply.

### 2.2. Research Methodology

The thickness of the oxide layers was measured using a Fischer Dualscope MP40 thickness gauge, which uses the Eddy-current method for measuring. Ten measurements were made on the sample length (with three repetitions), then the average value was calculated. The accuracy of the Dualscope device is 1 µm.

Morphology tests of the oxide layers were carried out with a Hitachi S-4700 scanning electron microscope with a Noran Vantage EDS system at magnifications of 50,000×, which enabled observation of the nanopore distribution. The layers of amorphous aluminum oxide do not allow the electrons diffracted during interaction of the microscope beam to be discharged. Therefore, the examined layers were previously sputtered with carbon. Three microscopic photographs were taken from different places on the oxide layers. The ImageJ 1.50i program was used for the computer analysis of the digitized microscopic photographs. The average surface stereological parameters were calculated using the appropriate procedures of computer image analysis. The calculations determined the porosity, the number of nanopores per unit area, and the size of the nanopores. The data was used to create histograms showing the distribution of the surface area of the nanopores.

Two liquids were used to measure the contact angle: polar (water) and non-polar (diiodomethane). Ten drops of each liquid were applied to the surface to be tested using a 0.5 μL micropipette. A camera was used to take photographs of each drop and the photographs were transferred to a computer. Measurement of the contact angle was carried out using software which enabled the selection of three drop points, and then automatic determination of the angle. The largest and smallest contact angles were rejected as extreme, the remaining eight contact angles served to calculate the mean contact angle for a particular sample. The surface-free energy was calculated using the contact angle values by means of the Owens-Wendt method. The Owens-Wendt method assumes that the value of surface-free energy is the sum of the dispersion γ_s_^d^ and polar γ_s_^p^ components. The relationship between these values is determined by the equation:

γ_s_ = γ_s_^d^ + γ_s_^p^ (1)

where γ_s_—surface-free energy of a solid, γ_s_^d^—dispersive component of the surface-free energy of the investigated material, γ_s_^p^—polar component of the surface-free energy of the investigated material.

The phase composition of the layers was determined by the standard method using an X-ray diffractometer from PANalytical, model X’Pert Pro MPD PW3040/60, using Cu Kα1 radiation and the HighScore computer program. During the test, the following measurement conditions were used: 30 kV lamp voltage, 10 mA lamp current, 0.02° 2Θ stepping angle, 0.005° 2Θ/s recording rate. The angle range of the performed diffractograms was 10°–80° 2Θ.

## 3. Results and Discussion

The averaged values of the oxide layer thickness measurements are presented in Table 2.

The tests show considerable differences in the thickness of the oxide layers resulting from the conditions of the anodizing process. The thickness of each layer depends on the density of the electric charge and the temperature of the electrolyte. With an increase in electric current density, while maintaining a constant process time and electrolyte temperature, the growth of each Al_2_O_3_ layer thickness is increased significantly. (Samples A, K; D, L). An increase in temperature causes a slight decrease in the layer thickness growth (Samples A, B, C, D; F, G, H; K, L). The influence of electrolyte temperature increase on reduction in the thickness of the Al_2_O_3_ layers can be explained by the increasing ability of secondary aluminum oxide dissolution in higher temperature electrolytes.

**Figure 1:**
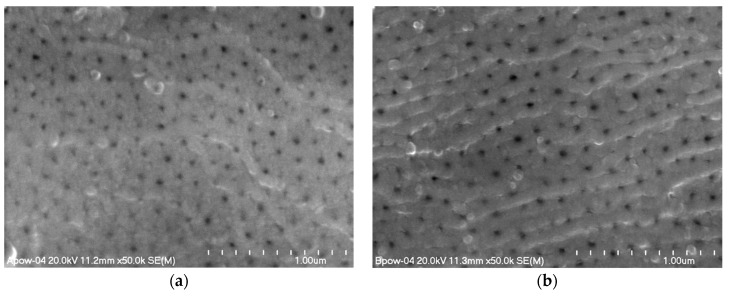

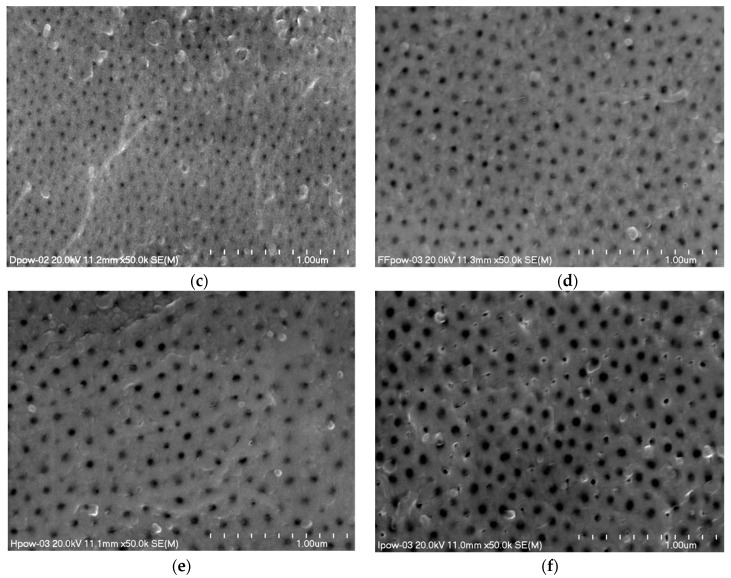
Images of surface morphology of oxide layer: (**a**) Sample A, (**b**) Sample B, (**c**) Sample D, (**d**) Sample F, (**e**) Sample H, (**f**) Sample I.

The morphology images of the oxide layers made using scanning electron microscopy show the porosity of the surface, characteristic of aluminum oxides, whose elements are nanopores. The porosity visible on the entire surface of the layers as seen in Figure 1 shows the effect of the columnar structure of the oxide. Figure 1 shows selected images of the surfaces of oxide layers showing the largest differences in the number and average size of the nanopores.

**Figure 2:**
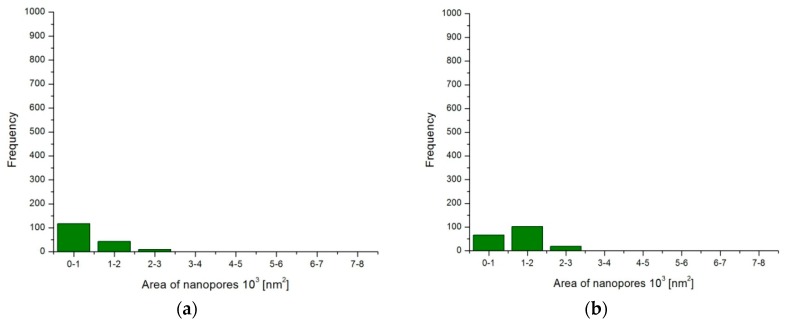

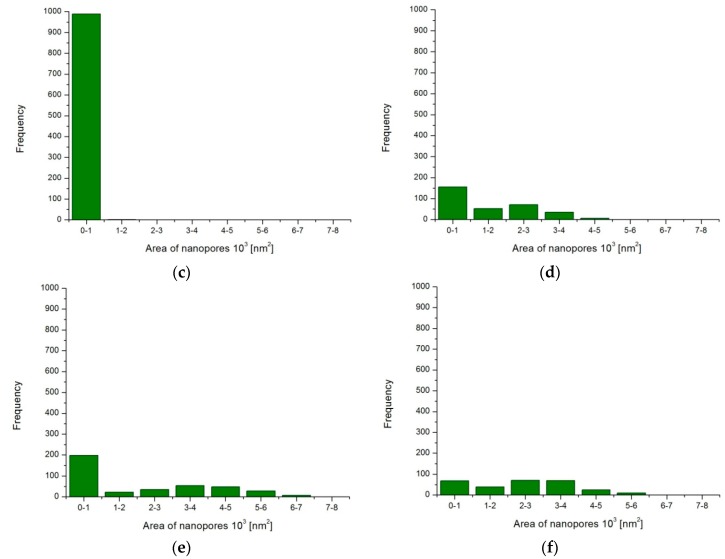
Histogram of occurrence of nanopores and their surfaces: (**a**) Sample A, (**b**) Sample B, (**c**) Sample D (smallest pores), (**d**) Sample F, (**e**) Sample H, (**f**) Sample I.

The microscopic photographic images of the layers subjected to computer analysis were processed using the same procedures. Based on the obtained data, histograms were prepared with the distribution of nanopore surfaces in individual layers as seen in Figure 2 and a table with the stereological parameters of the surface layers (Table 3).

The computer analysis of the layer images showed the effect of the anodizing parameters on the number and average size of nanopores. The anodic current density used during the process has a significant influence on the surface porosity of Al_2_O_3_ layers. A higher current density affects the speed of the process, which is directly related to the growth and shape of the oxide layer cells. The tests have shown that with an increase in the current of the process (Samples A, K; D, L), the pore diameter increases, while the fraction of open pore surface decreases. This happens independent of the electrolyte temperature. The temperature of the electrolyte has a similar effect on the porosity of the surface of the Al_2_O_3_ layers. Temperature growth in the vicinity of the anode causes a more porous layer. The more porous layer is visible mainly for higher values of current density and process time (Samples F, G, H; K, L).

Table 4 shows the contact angle values using distilled water and diiodomethane as the measuring liquid. Table 5 shows the contact angle values using α-bromonaphthalene and glycerin as the measuring liquid. Different values of contact angles for individual oxide layers were obtained depending on the production parameters. The highest contact angle value was obtained for Sample A, produced at the electrolyte temperature of 283 K, current density of 1 A/dm^2^, over a period of 20 min. The smallest contact angle was observed for Sample F anodized in the electrolyte at 298 K, current density 3 A/dm^2^, over a period of 60 min. It was observed that higher contact angle values were determined for the extreme values of current density and electrolyte temperatures.

Table 6 shows the surface-free energy values determined for the Al_2_O_3_ surface. The contact angle measurements were used to determine the surface-free energy. The Owens-Wendt method (according to Equation (1)) was employed in the calculations using the measurements of the deposited drop of polar and non-polar liquid.

The calculations show an inversely proportional dependence of contact angle values to surface-free energy. Surfaces with a low surface-free energy (contact angle >90°) are characterized by low surface wettability and can be used on the skin of aircraft and wind turbines. Such surfaces are also resistant to wear in sliding combinations.

**Figure 3:**
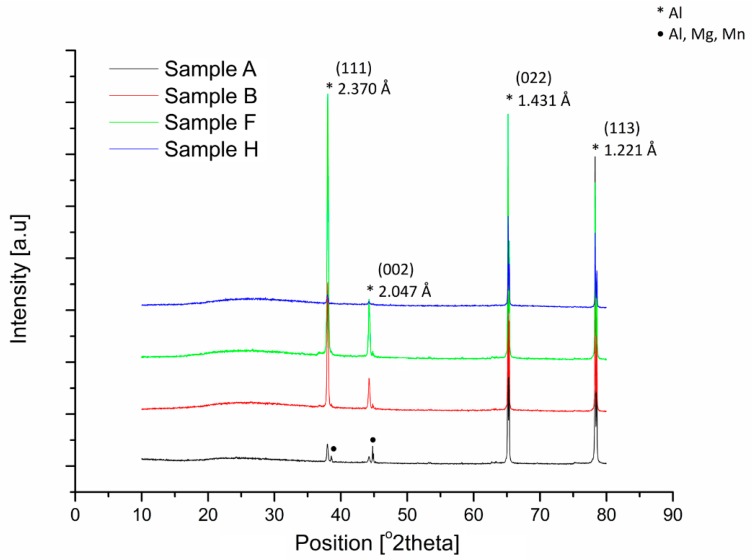
Phase analysis of selected oxide layers.

The phase structure of the Al_2_O_3_ layers obtained by the electrochemical method has aroused controversy over many years. Some researchers (J. D. Edwards, F. Keller) concluded that these oxide layers have an a γ-Al_2_O_3_ crystalline structure. Other researchers (J. J. Trilland, R. Tertian) concluded that the layers have a crystalline structure composed of a mixture of monohydrate and γ-Al_2_O_3_, while the barrier layer has an amorphous structure. Phase analysis conducted for selected oxide layers characterized by the greatest variations in the number and average size of nanopores showed that the Al_2_O_3_ layers produced by the electrochemical method are amorphous over their entire thickness, regardless of the conditions of their fabrication as seen in Figure 3, which is consistent with the results of other researchers [20].

On the X-ray diffraction pattern of the Al_2_O_3_ layers, strong reflections (the crystalline phase) belonging to aluminum (2.370 Å, 2.047 Å, 1.431 Å, 1.221 Å) are noticeable. In addition weaker reflections belonging to aluminum alloys with alloy admixtures of magnesium and manganese (2.335 Å) oxide layers are noticeable. In all cases, it can be seen that the background of the diffraction pattern increased in the range of 20°–45° 2Θ. An increase in the diffractogram background “amorphous halo” in the above-mentioned angular range is characteristic of the amorphous, non-reflective Al_2_O_3_ layers. The intensity of the raised diffractogram background was dependent on the thickness of the oxide layers. The penetration depth of X-ray radiation depends on the type of material being examined. At low angles of incidence, the X-rays penetrate only the uppermost layers of the sample. At higher angles of incidence, the X-rays penetrate deeper into the sample, which is why the X-rays gave an image of the material from the entire thickness of the sample, not just the uppermost layers.

**Figure 4:**
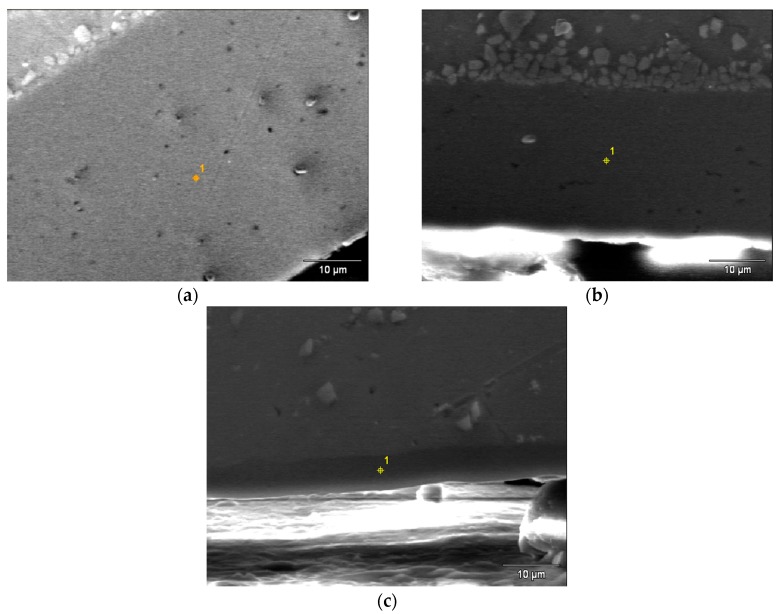
Measurement point of chemical composition of Al_2_O_3_ layers: (**a**) Sample H, (**b**) Sample K, (**c**) Sample A.

The chemical composition of aluminum oxide according to the stoichiometric calculations should be: 52.92% aluminum and 47.08% oxygen (atomic content). The analysis of the chemical composition of the Al_2_O_3_ layers obtained in a ternary electrolyte on the EN AW-5251 alloy substrate showed (Table 7) that in the middle zone of thicker layers, the chemical composition was similar to the stoichiometric calculations as seen in Figure 4a.

Tests carried out for thinner layers showed an increased aluminum content and reduced oxygen content as seen in Figure 4b,c. The reduction in aluminum content and the increase in oxygen content for thicker Al_2_O_3_ layers is the cause of the change in the stoichiometry of the layers resulting from the nature of the anodizing process.

## 4. Conclusions

Based on the conducted tests, it can be concluded that a change in the surface anodizing process parameters of the aluminum influences the morphology and surface-free energy of the oxide layers. The tests have shown that with an increase in the current of the process, the pore surface increases, while the number of pores decreases. The smallest values of surface-free energy were found in the layers produced at the extreme current and temperature parameters. The oxide layers showed mostly hydrophilic properties because only one sample produced in the electrolyte at 283 K, 1 A/dm^2^, in 20 min showed a contact angle that exceeded 90°. It was also found that the oxide layer surfaces with the lower proportion of porosity showed higher values of contact angle, which is in line with theory. On the basis of the wetting state, smooth surfaces correspond to Young’s equation, while porous surfaces can be modelled by the Wenzel equation or the Cassie–Baxter Equation [21].

## Figures and Tables

**Table 1:** Anodizing conditions.

Sample	Current Density j (A/dm^2^)	Process Time t (min)	Electrolyte Temperature T (K)
A	1	20	283
B	1	20	293
C	1	20	303
D	1	20	313
E	2	60	293
F	3	60	293
G	3	60	298
H	3	60	303
I	4	45	303
J	4	45	298
K	4	20	283
L	4	20	313

**Table 2:** List of oxide layer thicknesses.

Sample	Thickness (µm)	Deviation (µm)
A	6.5	0.6
B	5.6	0.4
C	5.3	0.7
D	3.3	0.6
E	36.6	1.1
F	52.9	1.7
G	51.4	1
H	50.2	1
I	53.5	1.5
J	53.5	2.5
K	26.3	1.5
L	23.6	0.6

**Table 3:** Stereological parameter values from computer image analysis.

Sample	Porosity (%)	Deviation (%)	Pore Density (Number of Pores/µm^2^)	Deviation (Number of Pores/µm^2^)	Average Area of Nanopores (nm^2^)	Deviation (nm^2^)
A	4.2	0.16	59.2	2.26	706.9	26.93
B	4.4	0.19	34.5	1.49	1275.2	55.07
C	4.8	0.20	83.9	3.50	568.6	23.69
D	3.3	0.11	182.8	6.11	183	6.10
E	5.7	0.21	66.7	2.46	858.1	31.61
F	8.2	0.38	59	2.73	1378.9	63.90
G	7.5	0.33	40.2	1.77	1849.1	81.36
H	14.6	0.61	72.9	3.01	2004.7	83.76
I	12.3	0.46	52.2	1.95	2358.7	88.22
J	11.5	0.46	55.2	2.21	2086.6	83.46
K	3.6	0.21	36.3	2.12	997.1	58.16
L	10.3	0,41	59.27	2.36	1744	69.42

**Table 4:** Contact angles of oxide layer for distilled water and diiodomethane.

Sample	Contact Angle (Distilled Water) (°)	Deviation (°)	Contact Angle (Diiodomethane) (°)	Deviation (°)
A	90.8	2.70	59.05	2.26
B	65.49	5.98	48.4	4.75
C	82.25	5.14	47.74	4.38
D	83.18	4.85	44.74	4.25
E	71.8	3.25	47.15	5.56
F	61.66	5.52	48.18	2.69
G	73.86	2.83	46.84	3.23
H	69.55	6.50	46.25	3.17
I	79.82	3.08	51.35	4.02
J	75.13	2.51	59.05	3.76
K	83.4	2.29	48.4	2.29
L	84.3	3.42	47.74	3.74

**Table 5:** Contact angles of oxide layer for α-bromonaphthalene and glycerin.

Sample	Contact Angle (α-Bromonaphthalene) (°)	Deviation (°)	Contact Angle (Glycerin) (°)	Deviation (°)
A	39.88	4.44	84.25	7.69
B	33.05	2.80	73.80	2.55
C	37.68	3.23	80.22	5.24
D	34.36	4.37	83.62	4.41
E	29.71	4.48	69.59	4.51
F	30.09	2.70	72.40	3.29
G	24.97	4.12	71.95	4.70
H	33.63	1.78	72.01	2.44
I	29.54	1.57	74.46	3.58
J	29.91	2.36	73.40	3.71
K	32.40	3.79	75.75	3.33
L	31.07	2.37	74.25	3.37

**Table 6:** Surface free energy values.

Sample	SFE Owens-Wendt (mJ/m^2^)
A	31.36
B	46.51
C	40.39
D	39.04
E	43.66
F	49.12
G	40.84
H	43.08
I	39.08
J	41.32
K	38.42
L	38.24

**Table 7:** Analysis of chemical composition of Al_2_O_3_ layers.

Sample	Atomic Aluminum Content (%)	Error of Aluminum Content (%)	Atomic Oxygen Content (%)	Error of Oxygen Content (%)
A	72.66	±0.39	26.88	±1.15
K	59.94	±0.31	39.94	±0.93
H	57.13	±0.29	42.36	±0.86

## References

[1] Davis J.R. (1993). Aluminum and Aluminum Alloys.

[2] Davis J.R. (1999). Corrosion of Aluminum and Aluminum Alloys.

[3] Diggle J.W., Downie T.C., Goulding C.W. (1969). Anodic oxide films on aluminum. Chem. Rev..

[4] Thompson G.E. (1997). Porous anodic alumina: Fabrication, characterization and applications. Thin Solid Films.

[5] Sulka G.D., Eftekhari A. (2008). Highly ordered anodic porous alumina formation by self-organized anodizing. Nanostructured Materials in Electrochemistry.

[6] Lee W., Ji R., Gosele U., Nielsch K. (2006). Fast fabrication of long-range ordered porous alumina membranes by hard anodization. Nat. Mater..

[7] Stepniowski W.J., Moneta M., Karczewski K., Michalska-Domanska M., Czujko T., Mol J.M., Buijnsters J.G. (2018). Fabrication of copper nanowires via electrodeposition in anodic aluminum oxide templates formed by combined hard anodizing and electrochemical barrier layer thinning. J. Electroanal. Chem..

[8] Sattler K. (2011). Handbook of Nanophysics/Functional Nanomaterials.

[9] Liu P., Singh V.P., Rajaputra S. (2010). Barrier layer nonuniformity effects in anodized aluminum oxide nanopores on ITO substrates. Nanotechnology.

[10] Fratila-Apachitei L.E., Tichelaar F.D., Thompson G.E., Terryn H., Skeldon P., Duszczyk J., Katgerman L. (2004). A transmission electron microscopy study of hard anodic oxide layers on AlSi(Cu) alloys. Electrochim. Acta.

[11] Runge J.M. (2018). The Metallurgy of Anodizing Aluminum—Connecting Science to Practice.

[12] Zhang L., Cho H.S., Li F., Metzger R.M., Doyle W.D. (1998). Cellular growth of highly ordered porous anodic films on aluminium. J. Mater. Sci. Lett..

[13] Jia Y., Zhou H., Luo P., Luo S., Chen J., Kuang Y. (2006). Preparation and characteristics of well-aligned macroporous films on aluminum by high voltage anodization in mixed acid. Surf. Coat. Technol..

[14] Kubica M., Skoneczny W., Bara M. (2018). Analysis of Al_2_O_3_ Nanostructure Using Scanning Microscopy. Scanning.

[15] Bara M., Skoneczny W., Hajduga M. (2009). Ceramic-graphite surface layers obtained by the duplex method on an aluminium alloy substrate. Chem. Process. Eng..

[16] Wang Q., Zhang B.W., Qu M.N., Zhang J.Y., He D.Y. (2008). Fabrication of superhydrophobic surfaces on engineering material surfaces with stearic acid. Appl. Surf. Sci..

[17] Zhang X., Shi F., Niu J., Jiang Y.G., Wang Z.Q. (2008). Superhydrophobic surfaces: From structural control to functional application. J. Mater. Chem..

[18] Sooksaena P., Chulasinonta O., Janmata P., Thovasakula W. (2017). Chemical treatment on aluminum alloy for hydrophobic surfaces. Mater. Today-Proc..

[19] Skoneczny W., Niedźwiedź M., Bara M. (2018). The Effect of Production Parameters of Oxide Layers on Their Nanostructure, Nanomorphology, and Surface Free Energy. Appl. Sci..

[20] Korzekwa J., Tenne R., Skoneczny W., Dercz G. (2013). Two-step method for preparation of Al_2_O_3_ /IF-WS_2_ nanoparticles composite coating. Phys. Status Solidi (A) Appl. Mater. Sci..

[21] Whyman G., Bormashenko E., Stein T. (2008). The rigorous derivation of Young, Cassie–Baxter and Wenzel equations and the analysis of the contact angle hysteresis phenomenon. Chem. Phys. Lett..

